# Overexpression of *flv3* improves photosynthesis in the cyanobacterium *Synechocystis* sp. PCC6803 by enhancement of alternative electron flow

**DOI:** 10.1186/s13068-014-0183-x

**Published:** 2014-12-31

**Authors:** Tomohisa Hasunuma, Mami Matsuda, Youhei Senga, Shimpei Aikawa, Masakazu Toyoshima, Ginga Shimakawa, Chikahiro Miyake, Akihiko Kondo

**Affiliations:** Organization of Advanced Science and Technology, Kobe University, 1-1 Rokkodai, Nada, Kobe, 657-8501 Japan; Precursory Research for Embryonic Science and Technology (PRESTO), Japan Science and Technology Agency, 3-5 Sanbancho, Chiyoda-ku, Tokyo, 102-0075 Japan; Department of Chemical Science and Engineering, Graduate School of Engineering, Kobe University, 1-1 Rokkodai, Nada, Kobe, 657-8501 Japan; Kobe University Center for Inland Sea, 1-1 Rokkodai, Nada, Kobe, 657-8501 Japan; Department of Biological and Environmental Science, Faculty of Agriculture, Graduate School of Agricultural Science, Kobe University, 1-1 Rokkodai, Nada, Kobe, 657-8501 Japan; Core Research for Evolutional Science and Technology (CREST), Japan Science and Technology Agency, 3-5 Sanbancho, Chiyoda, Tokyo, 102-0075 Japan; Biomass Engineering Program, RIKEN, 1-7-22 Suehiro, Tsurumi-ku, Yokohama, 230-0045 Japan

**Keywords:** *Synechocystis* sp. PCC6803, *flv3*, Alternative electron flow, Carbon metabolism, Photosynthesis, Metabolite profiling

## Abstract

**Background:**

To ensure reliable sources of energy and raw materials, the utilization of sustainable biomass has considerable advantages over petroleum-based energy sources. Photosynthetic algae have attracted attention as a third-generation feedstock for biofuel production, because algae cultivation does not directly compete with agricultural resources, including the requirement for productive land and fresh water. In particular, cyanobacteria are a promising biomass feedstock because of their high photosynthetic capability.

**Results:**

In the present study, the expression of the *flv3* gene, which encodes a flavodiiron protein involved in alternative electron flow (AEF) associated with NADPH-coupled O_2_ photoreduction in photosystem I, was enhanced in *Synechocystis* sp. PCC6803. Overexpression of *flv3* improved cell growth with corresponding increases in O_2_ evolution, intracellular ATP level, and turnover of the Calvin cycle. The combination of *in vivo*^13^C-labeling of metabolites and metabolomic analysis confirmed that the photosynthetic carbon flow was enhanced in the *flv3*-overexpressing strain.

**Conclusions:**

Overexpression of *flv3* improved cell growth and glycogen production in the recombinant *Synechocystis* sp. PCC6803. Direct measurement of metabolic turnover provided conclusive evidence that CO_2_ incorporation is enhanced by the *flv3* overexpression. Increase in O_2_ evolution and ATP accumulation indicates enhancement of the AEF. Overexpression of *flv3* improves photosynthesis in the *Synechocystis* sp. PCC6803 by enhancement of the AEF.

**Electronic supplementary material:**

The online version of this article (doi:10.1186/s13068-014-0183-x) contains supplementary material, which is available to authorized users.

## Background

Environmental concerns associated with the continued depletion of oil reserves and greenhouse gas emissions have prompted governments to promote research on environmentally benign and sustainable fuels towards realizing energy independence. In particular, utilization of biomass as the starting material for fuel production has recently attracted considerable attention [1,2]. It is anticipated that the use of biofuels as an alternative to fossil fuels will help reduce CO_2_ accumulation through reduced emissions and recycling of the CO_2_ that is released when biofuel is combusted as a transport fuel.

Photosynthetic algae are of increasing interest as a renewable source of biomass for the sustainable production of biofuels [3-5], because algal feedstocks do not directly compete with agriculture, as they require no productive land. Cyanobacteria are particularly attractive, because their photosynthetic and biomass production rates are higher than those of terrestrial plants [3,6]. Several cyanobacterial species have the ability to store large amounts of energy-rich glycogen that can be utilized for the production of biofuels, including bio-ethanol [7,8]. In addition, because cyanobacteria are prokaryotes, their metabolic processes are more readily amenable to genetic modifications designed to enhance photosynthetic activity than are those of eukaryotic algae.

In the phototrophic metabolism, carbon assimilation through the Calvin cycle is coupled with consumption of ATP and NADPH. Cyanobacterial cell growth rate is dependent on the intensity of light with the exception of photoinhibition and photooxidative stress, as it drives photosynthetic electron transport and the production of ATP and NADPH. ATP is required not only for turnover of the Calvin cycle, but also for the CO_2_ concentrating mechanism, which serves to increase the intracellular CO_2_ concentration [9-11]. Furthermore, ATP is required for glycogen biosynthesis in photosynthetic cells [12], meaning that a continuous supply of ATP is necessary for a higher rate of photosynthesis in cyanobacteria.

ATP is produced by ATP synthase via a proton gradient (ΔpH) across the thylakoid membrane. ΔpH is mainly generated by the photosynthetic linear electron flow (LEF) pathway, although phototrophs, including cyanobacteria, have also developed a large arsenal of alternative electron flow (AEF) pathways, which assist in modulating the ATP/NADPH ratio in response to metabolic demand [13,14]. O_2_-dependent electron flow, termed the Mehler pathway or water-water cycle (WWC), is one AEF pathway that helps generate a pH gradient [15-17]. In the Mehler pathway, electrons extracted from water by photosystem II (PSII) are used to generate water by the photoreduction of O_2_ coupled with the oxidation of NADPH in photosystem I (PSI).

In cyanobacteria, flavodiiron (Flv) proteins possess an NAD(P)H:flavin oxidoreductase module [18]. The cyanobacteria *Synechocystis* sp. PCC6803 has four genes encoding Flv proteins (Flv1, Flv2, Flv3, and Flv4). The results of an *in vitro* study with an *flv3* mutant provided evidence that Flv3 functions as an NAD(P)H:oxygen oxidoreductase [18]. A subsequent *in vivo* study with *Δflv1* and *Δflv3* mutants of *Synechocystis* sp. PCC6803 confirmed that Flv1 and Flv3 are involved in the photoreduction of O_2_ to H_2_O in the Mehler reaction [19]. Under fluctuating light conditions, the growth and photosynthesis of *Δflv1* and *Δflv3* mutants of *Synechocystis* sp. PCC6803 are arrested [20].

In the present study, a recombinant *flv3*-overexpressing *Synechocystis* strain (Flv3ox) was constructed to examine the effects of *flv3* overexpression on the photosynthetic ability of *Synechocystis* sp. PCC6803. Enhancement in the AEF pathway through the regeneration of NADP^+^ improved ATP accumulation in the Flv3ox cell. Recently, we developed an analytical method to directly measure the turnover of metabolic intermediates in cyanobacteria [21]. The combination of *in vivo*^13^C-labeling of metabolites and metabolome analysis using mass spectrometry (MS) enables kinetic visualization of carbon assimilation in central metabolic pathways, such as the Calvin cycle, glycolysis, and glycogen biosynthesis. Here, the dynamic metabolic profiling approach was used to reveal the underlying mechanisms of the enhancement of the photosynthetic carbon flow in the Flv3ox strain.

## Results

### Construction of an *flv3*-overexpressing recombinant strain

**Figure 1:**
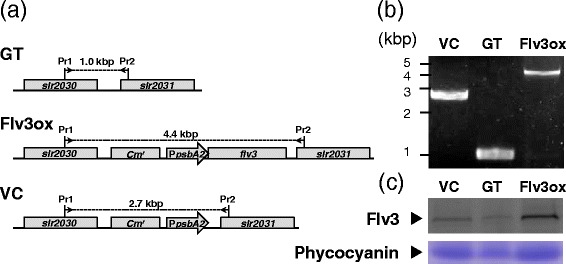
**Molecular characterization of the parental (GT),** ***flv3*** **-overexpressing (Flv3ox), and vector control (VC) strains. (a)** Genome structure around integration site in glucose-tolerant (GT), Flv3ox, and vector control (VC) strains. Pr1 and Pr2 indicate the positions of the primer used for the PCR screening. *Cm* ^*r*^, chloramphenicol resistance cassette; *PpsbA2*, *psbA2* promoter. **(b)** Genomic PCR analysis of GT, Flv3, and VC strains using Pr1 and Pr2. **(c)** Immunoblot analysis of Flv3 protein in GT, Flv3ox, and VC cells. Total soluble protein extracted from 1.6 mg DCW *Synechocystis* cells was reacted with anti-Flv3 antibody after separation on SDS-polyacrylamide gel. Phycocyanin was detected by staining with Coomassie brilliant blue. The amount of protein loaded on the gel is 75 μg (GT), 63 μg (Flv3ox), and 70 μg (VC).

To enhance *flv3* expression in *Synechocystis* sp. PCC6803, we constructed the transformation vector pTCP2031V-flv3, which contained *flv3* linked to the *psbA2* promoter between the *slr2030* and *slr2031* genes, which acted as anchoring regions for site-specific integration into the *Synechocystis* genome through homologous recombination (Figure 1a). A glucose-tolerant (GT) strain of *Synechocystis* sp. PCC6803 was transformed with pTCP2031V-flv3 to yield strain Flv3ox. The chromosomal integration of *flv3* was confirmed by genomic PCR (Figure 1b). A vector control (VC) strain, in which the chloramphenicol resistance cassette was inserted into the genome of GT, was constructed with an empty vector pTCP2031V. Immunoblot analysis showed higher levels of Flv3 protein in the Flv3ox strain compared to the parental GT and vector control strains (Figure 1c).

### Cell growth and O_2_ evolution by Flv3ox

**Figure 2:**
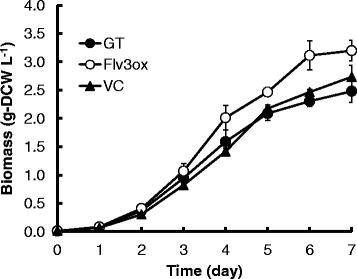
**Time course of cellular biomass in GT, Flv3ox, and VC cultivated under 120 μmol photons m** ^**-2**^ **s** ^**-1**^ **light intensity and 1% CO**_**2**_ **conditions.** The values are the mean ± SD of ten different measurements.

**Figure 3:**
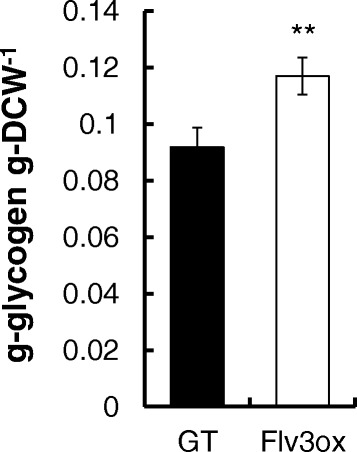
**Glycogen content in GT and Flv3ox cultivated under 120 μmol photons m** ^**-2**^ **s** ^**-1**^ **light intensity and 1% CO**_**2**_ **conditions.** The values are the mean ± SD of six different measurements. Statistical significance was determined using the Student’s *t*-test (^**^ *P* < 0.01).

Figure 2 shows the growth of strain GT, Flv3ox, and VC cells cultivated under continuous irradiation with 120 μmol photons m^-2^ s^-1^ light intensity and 1% (v/v) CO_2_ at 30°C. Under these conditions, Flv3ox showed a higher growth rate than the GT and VC strains. After a 7-day cultivation, the cell concentration of Flv3ox reached 3.20 g-dry cell weight (DCW) L^-1^ and that of GT was 2.48 g-DCW L^-1^. According to statistical analysis, the biomass concentration of Flv3ox was significantly different from that of GT and VC (Additional file 1). VC demonstrated a similar cell growth ability as GT. Measurements of glycogen showed that Flv3ox had a higher glycogen content (0.117 g-glycogen g-DCW^-1^) than that of GT (0.091 g-glycogen g-DCW^-1^) (Figure 3).

**Figure 4:**
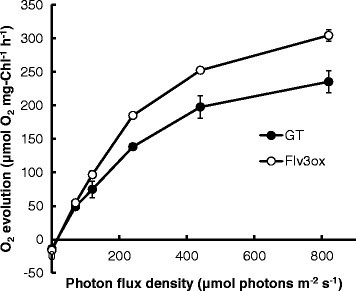
**Light response curves for O**_**2**_ **evolution rate in GT and Flv3ox cells.** When the OD_750_ reached approximately 4.5, the cells were applied for the photosynthesis analysis. The values are the mean ± SD of three measurements.

The light dependence of the O_2_ evolution rate by the two strains of *Synechocystis* cells was evaluated with an O_2_ electrode system (Figure 4). Flv3ox exhibited a higher O_2_ evolution rate than that of GT.

### Metabolic analysis of *Synechocystis* cells

**Figure 5:**
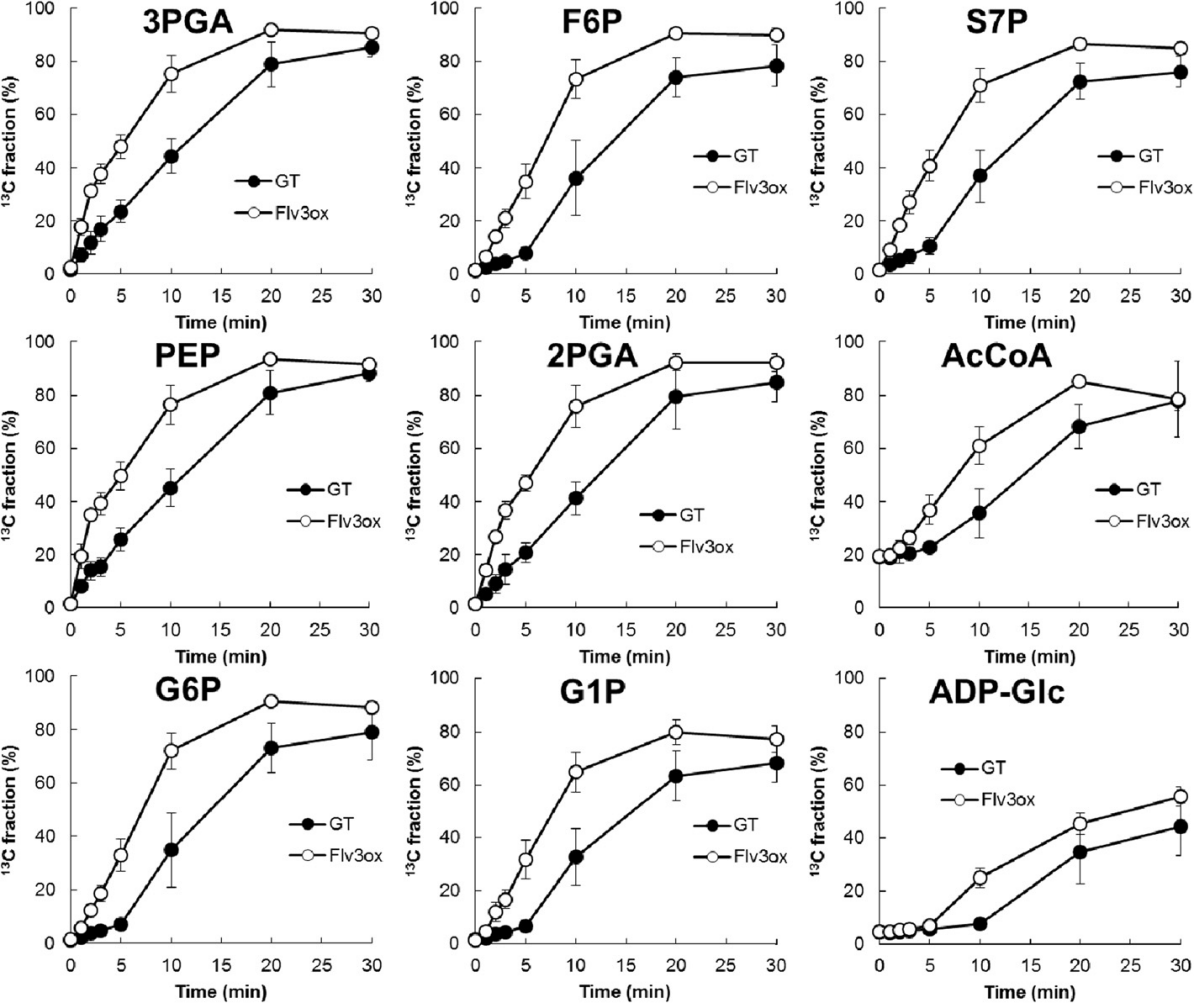
**Time course of the** ^**13**^ **C fraction for various metabolites in GT and Flv3ox cells cultivated under 120 μmol photons m** ^**-2**^ **s** ^**-1**^ **light intensity and 1% CO**_**2**_ **conditions.** The values are the mean ± SD of at least five different experiments.

Photosynthetic electron flow generates a proton gradient across the cyanobacterial thylakoid membrane, driving the ATP synthesis necessary for carbon assimilation. Here, the effect of *flv3* overexpression on intracellular carbon metabolism was investigated using a dynamic profiling technique [21] that measures the turnover of metabolic intermediates in cyanobacterial cells. The kinetic measurements were performed by the combination of an *in-vivo*^13^C-labeling technique and mass isotopomer detection with capillary electrophoresis coupled to MS (CE/MS) for the efficient separation of highly polar molecules. To assess metabolic turnover, extracted intracellular metabolites were analyzed after labeling for between 1 and 30 min (Figure 5). The ^13^C fraction, which was defined as the ratio of ^13^C to total carbon in each metabolite, was calculated from mass isotopomer distributions.

**Figure 6:**
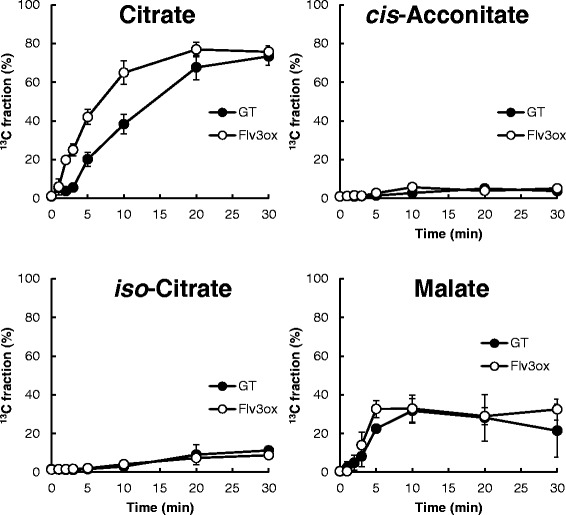
**Time course of the** ^**13**^ **C fraction of metabolites involved in the citrate cycle in GT and Flv3ox cells cultivated under 120 μmol photons m** ^**-2**^ **s** ^**-1**^ **light intensity and 1% CO**_**2**_ **conditions.** The values are the mean ± SD of at least five different experiments.

Ribulose-1,5-bisphosphate (RuBP) carboxylase/oxygenase (Rubisco) catalyzes the first major step of carbon fixation in the Calvin cycle to produce 3-phosphoglycerate (3PGA) from CO_2_ and RuBP. For Flv3ox, the ^13^C fraction of 3PGA reached a maximum of 92% after 20 min of labeling, whereas a maximum of 85% was observed after 30 min of labeling for GT (Figure 5). The overexpression of *flv3* resulted in an increase in ^13^C-labeling rate of metabolites involved in the Calvin cycle, including 3PGA, fructose-6-phosphate (F6P), and sedoheptulose-7-phosphate (S7P). Flv3ox also displayed a higher turnover rate of metabolites involved in glycolysis and glycogen biosynthesis, such as phospho*enol*pyruvate (PEP), 2-phosphoglycerate (2PGA), acetyl-CoA (AcCoA), glucose-6-phosphate (G6P), glucose-1-phosphate (G1P), and ADP-glucose (ADP-Glc). These results clearly showed that the enhancement of *flv3* expression accelerates the photosynthetic carbon assimilation rate. In addition, *flv3* overexpression resulted in an increase in the turnover rate of citrate, while the ^13^C fraction of other metabolites involved in the citrate cycle, including *cis*-acconitate, isocitrate, and malate, was almost the same between Flv3ox and GT (Figure 6).

Table 1 shows the pool sizes of intracellular metabolites in the two strains of cyanobacterial cells. Flv3ox accumulated 0.207 μmol g-DCW^-1^ ATP, while ATP level in GT was lower than the quantification limit (0.01 μmol g-DCW^-1^) of the CE/MS analysis. There was no significant difference in NADPH level between Flv3ox and GT. NADP^+^ was slightly (1.6-fold) increased by the *flv3* overexpression. The levels of F6P, G6P, and S7P in Flv3ox were similar to those in GT, while an increase in 3PGA and a decrease in RuBP were caused by the *flv3* overexpression. Flv3ox showed higher amounts of ADP-Glc and lower G1P than GT. 2PGA and PEP were also increased by the *flv3* overexpression.

**Table 1:** **Metabolite pools in GT and Flv3ox cells cultivated under 120 μmol photons m** ^**-2**^ **s** ^**-1**^ **light intensity and 1% CO**_**2**_ **conditions**

	**Concentration (μmol g-DCW** ^**-1**^ **)**	
**Metabolite**	**GT**	**Flv3ox**
3PGA	2.267 ± 0.061	3.157 ± 0.191^**^
F6P	0.071 ± 0.007	0.069 ± 0.011
S7P	0.096 ± 0.003	0.094 ± 0.006
RuBP	0.089 ± 0.006	0.026 ± 0.005^**^
G6P	0.116 ± 0.015	0.138 ± 0.023
G1P	0.101 ± 0.016	0.020 ± 0.003^**^
ADP-Glc	0.080 ± 0.010	0.115 ± 0.004^**^
PEP	1.134 ± 0.039	1.562 ± 0.100^**^
2PGA	0.282 ± 0.008	0.401 ± 0.023^**^
AcCoA	0.075 ± 0.006	0.075 ± 0.015
ATP	< 0.01	0.207 ± 0.021
NADPH	0.001 ± 0.000	0.003 ± 0.002
NADP^+^	0.495 ± 0.012	0.549 ± 0.021^*^

Values are the mean ± SEM of nine different measurements. Statistical significance was determined using the Student’s *t*-test (^*^ *P* < 0.05, ^**^ *P* < 0.01).

## Discussion

The overexpression of *flv3* improves the cell growth of *Synechocystis* sp. PCC6803, as observed by the increase in the carbon assimilation rate as well as O_2_ evolution activity in Flv3ox cells. In Figure 5, an increase in the flux of photosynthetic carbon metabolism in Flv3ox was clearly demonstrated using a dynamic metabolic profiling approach developed by our group. So far, there has been no report that the improvement of photosynthetic CO_2_ incorporation was directly observed in recombinant cyanobacterial strains. When metabolism is in a dynamic steady state *in vivo*, metabolites are replaced with newly synthesized compounds at a constant rate, and the total amount of each metabolite remains unchanged. Thus, in order to understand metabolic flux, the turnover of metabolic intermediates should be examined directly. The measurement of metabolic turnover using this *in vivo*^13^C-labeling assay has provided conclusive evidence that CO_2_ incorporation is enhanced by *flv3* overexpression in *Synechocystis* cells.

**Figure 7:**
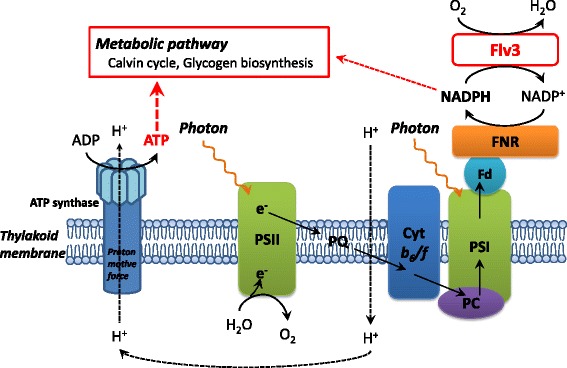
**Schematic representation of the AEF and ATP synthesis in** ***Synechocystis*** **cells.**

In the photosynthetic electron transport chain, electrons extracted from H_2_O in PSII are transferred to NADP^+^ to produce NADPH in PSI (Figure 7). Figure 4 demonstrated that the photosynthetic O_2_ evolution was improved by the enhancement of *flv3* expression. Because Flv3 regenerates NADP^+^ with the photoreduction of O_2_, overexpression of *flv3* in *Synechocystis* cells would increase photosynthetic electron transport.

In general, the energy budgets of phototrophs are dependent on the ratio of protons pumped across the thylakoid membrane to electrons passed through photosynthetic electron transfer complexes, which produce ATP and NADPH used in cellular biochemical pathways [22]. CO_2_ fixation through the Calvin cycle is the main ATP and NADPH sink in autotrophic metabolism, requiring an ATP/NADPH ratio of 1.5, whereas the ratio generated by the LEF pathway is 1.28 [13]. AEF pathways contribute to the balancing of ATP/NADPH by supplying additional ATP [14]. For example, the Mehler reaction generates a proton gradient across the thylakoid membranes, leading to ATP generation, but the net production of reduced NADPH is not expected [16]. Although the physiological function of the Mehler reaction in cyanobacteria is not fully understood, here, increased expression of the *flv3* gene in *Synechocystis* promoted NADP^+^ regeneration coupled with O_2_ photoreduction in the Mehler reaction, leading to a higher photosynthetic electron transport rate and increased ATP supply, as shown in Figure 4 and Table 1. Enhancement in the intracellular ATP level led to a higher ATP/NADPH ratio in the recombinant strain. The increased ATP supply would be coupled with the cell growth and improved carbon metabolic flux through the Calvin cycle, sugar phosphate pathway, and glycogen biosynthesis.

The overexpression of *flv3* increased the glycogen content in *Synechocystis* cells (Figure 3). Glycogen, an α-1,6-branched α-1,4-glucan, is the primary storage polysaccharide in cyanobacteria and is biosynthesized from F6P via G6P, G1P, and ADP-Glc as intermediates [12]. As ADP-Glc is produced from G1P and ATP by G1P adenyltransferase, the enhanced supply of ATP by *flv3* overexpression would lead to increased glycogen production. Increase in ADP-Glc and decrease in G1P by the *flv3* overexpression, which are demonstrated in Table 1, would indicate enhanced conversion of G1P to ADP-Glc.

Flv1 and Flv3 are essential for photoreduction of O_2_ in *Synechocystis* sp. PCC6803 [19]. Allahverdiyeva *et al.* reported that the growth and photosynthesis of *Δflv1* and *Δflv3* mutants was arrested in fluctuating light, although Flv1 and Flv3 proteins were dispensable under constant-light conditions [20]. Flv1 and Flv3 were proposed to form a heterodimer [23], while Flv3 alone is sufficient for the O_2_ photoreduction reaction *in vitro* [19]. The observed difference in the phenotypes of *Δflv1* and *Δflv3* mutants suggests that a homodimer of Flv3 exhibits NADPH-dependent O_2_ reduction *in vivo* under excess-light stress conditions [24]. Zhang *et al.* reported that *flv3* transcripts were markedly higher than those of *flv1* in wild-type *Synechocystis* under both 3% CO_2_ and ambient CO_2_ condition [25]. Our present results in an *flv3*-overexpressing strain of *Synechocystis* raise the possibility that the Flv3 protein can function alone, since overexpression of an *flv3* increased the level of ATP and flux of carbon assimilation pathways.

*Synechocystis* sp. PCC6803 is one of the most widely used species in studies of photosynthetic bacteria because of its unicellular morphology and fully sequenced genome [26]. In addition, the natural transformability of this strain has allowed for the establishment of reliable genome manipulation techniques, which enables the easy modification of metabolic pathways. This has been followed by systems biology approaches, such as multi-omics, dynamic metabolic profiling, and flux balance analyses, for gaining insight into cellular metabolic processes [5,27]. A systems biology approach is needed to exploit cyanobacteria as cell factories for the production of fuels and commodity chemicals through metabolic engineering. Our present findings suggest that the ATP required for the synthetic pathway could potentially be supplied through an enhanced AEF pathway to further increase the efficiency of cyanobacterial cell factories.

## Conclusions

Overexpression of the *flv3* gene, encoding an NAD(P)H:oxygen oxidoreductase, enhanced photosynthetic electron transport and ATP supply through the regeneration of NADP^+^ in the recombinant *Synechocystis* sp. PCC6803. We also observed improvement of CO_2_ fixation and carbon assimilation in central metabolic pathways such as the Calvin cycle and glycogen biosynthesis in the *flv3*-overexpressing strain by the combination of *in vivo*^13^C-labeling of metabolites and metabolome analysis, which would be due to the enhancement in the intracellular ATP level. The *flv3* overexpression improved not only the cell growth but also glycogen production in the *Synechocystis*, which would be a promising approach for the development of cyanobacterial biofuel production.

## Methods

### Strains and culture conditions

A GT strain of *Synechocystis* sp. PCC6803 [28], an *flv3*-overexpressing strain (Flv3ox), and its vector control (VC) strain were grown in BG11 liquid medium [29]. Cells were pre-cultivated in 500-mL flasks containing BG11 medium in an NC350-HC plant chamber (Nippon Medical and Chemical Instruments Co., Ltd., Osaka, Japan) under continuous irradiation at 50 μmol white light photons m^-2^ s^-1^ and 0.04% (v/v) CO_2_ condition with 100-rpm agitation at 30°C. Cultivation was performed in a closed double-deck flask, which had a first stage containing 50 mL of 2 M NaHCO_3_/Na_2_CO_3_ buffer with the appropriate pH to obtain the desired CO_2_ concentration of 1% (v/v), and a second stage containing 70 mL BG11 culture medium [30,31]. Pre-cultured cells were inoculated into the fresh BG11 medium at a biomass concentration of 1.26 mg DCW L^-1^ (the optical density at 750 nm [OD_750_] was 0.04) and cultivated under continuous irradiation at 50 or 120 μmol white light photons m^-2^ s^-1^ and 1% (v/v) CO_2_ with 100-rpm agitation at 30°C. Light intensity was measured in the middle of the medium using an LI-250A light meter equipped with an LI-190SA quantum sensor (LI-COR, Lincoln, NE). Cell density in the medium was determined as DCW, as a linear correlation was observed between DCW and optical density measured at OD_750_ using a UV mini spectrophotometer (Shimadzu, Kyoto Japan). For plate cultures, BG-11 was solidified using 1.5% (w/v) agar (BD Biosciences, San Jose, CA) and incubated in air at 30°C under continuous white light irradiation (50 to 70 μmol photons m^-2^ s^-1^).

### Construction of recombinant strains

The *flv3* (*sll0550*) coding region was amplified from *Synechocystis* sp. PCC6803 genomic DNA by PCR using the primer set 5′-GGAATTATAACCATAATGTTCACTACCCCCCTCCC-3′ and 5′-GGCATGGAGGACATATTAGTAATAATTGCCGACTT-3′. The resulting 1.7-kb fragment was integrated into *Nde*I-digested pTCP2031V [32] using an In-Fusion HD Cloning Kit (Takara Bio, Shiga, Japan) to yield pTCP2031-flv3. The GT strain was transformed with pTCP2031V-flv3 using a previously described method [33] to yield strain Flv3ox. Also, the plasmid pTCP2031V was introduced into the GT strain to yield the VC strain. Colonies resistant to 34 μg mL^-1^ chloramphenicol were selected, and isolation of a single colony was repeated three times. The chromosomal integration of *flv3* and plasmid-derived sequence was confirmed by PCR using the specific primers, Pr1 (5′-GGTAGTGGGCAATGCTGTAGAACAAGCGTTTGAGC-3′) and Pr2 (5′-CGGTAATTCTTGGCGCAATTGACGGTCAAAATAAC-3′). pTCP2031V was a kind gift from Professor M. Ikeuchi (University of Tokyo, Japan).

### Immunoblot analysis

Cellular proteins were extracted from 1.6 mg DCW *Synechocystis* cells with 50 μL of 5% lithium lauryl sulfate containing 75 mM dithiothreitol by sonication in a Biorupter UCD-200 bath sonicator (Cosmo Bio, Tokyo, Japan). After centrifugation of the mixture at 15,000 *g* for 5 min at 4°C, 10 μL of the extracts were subjected to sodium dodecyl sulfate-polyacrylamide gel electrophoresis on 12% and 18% polyacrylamide/6 M urea gels in a Tris/MES system [34]. After electrophoresis, proteins separated on the 12% gel were blotted onto a polyvinylidene fluoride membrane, reacted with rabbit anti-Flv3 antibody, which was kindly provided by Dr. H. Yamamoto (Kyoto University, Japan), and visualized with 5-bromo-4-chloro-3-indolyl-phosphate/nitroblue tetrazolium after incubation with alkaline phosphatase-conjugated Goat Anti-Rabbit IgG antibody (Promega, Madison, WI). Proteins separated on the 18% gel were stained with Coomassie brilliant blue R-250 (Nacalai Tesque, Inc., Kyoto, Japan). Protein concentrations were determined by using the QuantiPro BCA assay kit (Sigma-Aldrich, St. Louis, MO), with bovine serum albumin as the standard.

### Metabolite analysis

Cyanobacterial cells equivalent to 5 mg DCW were collected from the cultivation flasks and immediately filtered using 1-μm pore size polytetrafluoroethylene (PTFE) filter disks (Omnipore; Millipore, Billerica, MA). After washing the filter disks with 20 mM ammonium carbonate pre-chilled to 4°C, the cells retained on the filters were immediately placed into 2 mL pre-cooled (-30°C) methanol containing 37.38 μM L-methionine sulfone and 37.38 μM piperazine-1,4-bis(2-ethanesulfonic acid) as internal standards for mass analysis. Intracellular metabolites were extracted according to a previously reported method [21], with minor modification. The cells were suspended by vortexing, and then 0.5 mL of the cell suspension was mixed with 0.2 mL of pre-cooled (4°C) water and 0.5 mL of chloroform. After vortexing for 30 s, phase separation of aqueous and organic layers was performed by centrifugation at 14,000 *g* for 5 min at 4°C. 500 μL of the aqueous layer was filtered with a Millipore 5 kDa cut-off membrane for the removal of solubilized proteins. After evaporation of the aqueous-layer extracts under vacuum using a FreeZone 2.5 Plus freeze dry system (Labconco, Kansas City, MO), the dried metabolites were dissolved in 20 μL of Milli-Q water. The metabolites were analyzed using a CE/MS system (CE, Agilent G7100; MS, Agilent G6224AA LC/MSD TOF; Agilent Technologies, Palo Alto, CA) controlled by MassHunter Workstation Data Acquisition software (Agilent Technologies), as described previously [21].

### ^13^C-labeling experiment

To analyze metabolic turnover in cyanobacteria, *in vivo*^13^C-labeling was performed according to a previously reported method [21]. Briefly, *Synechocystis* cells were cultivated in BG11 medium under 120 μmol photons m^-2^ s^-1^ light intensity and 1% (v/v) CO_2_ conditions at 30°C. When the OD_750_ reached approximately 4.5, 5 mg DCW of cells were filtered and resuspended at an OD_750_ of 1.0 in BG11 medium containing 25 mM NaH^13^CO_3_. After labeling for 1 to 30 min, cells were collected by filtration and processed as described for the metabolite analysis. Extracted intracellular metabolites were analyzed using CE/MS. Mass spectral peaks of biological origin were identified manually by searching for mass shifts between the ^12^C and ^13^C mass spectra.

The ^13^C fraction of metabolites was calculated as described previously [35]. The relative isotopomer abundance (*m*_*i*_) for each metabolite in which *i*^13^C atoms are incorporated is calculated by the following equation:

$$ {m}_i\left(\%\right)=\frac{M_i}{{\displaystyle \sum_{j=0}^n{M}_j}}\times 100 $$

where *M*_*i*_ represents the isotopomer abundance for each metabolite in which *i*^13^C atoms are incorporated.

The ^13^C fraction of the metabolite possessing *n* carbon atoms is calculated by the following:

$$ {}^{13}\mathrm{C}\ \mathrm{fraction}\ \left(\%\right)={\displaystyle \sum_{i=1}^n\frac{i\times {m}_i}{n}} $$

### Glycogen measurement

When the OD_750_ of *Synechocystis* cultures reached approximately 4.5, cells were harvested using PTFE filter disks, as described above. After washing the filters with 20 mM ammonium carbonate, the cells on the filter were immediately frozen in liquid nitrogen, and then freeze-dried using a Labconco freeze dryer. After glycogen was extracted from the cells, the glycogen content was determined by high performance liquid chromatography (HPLC) (Shimadzu) using a size-exclusion HPLC column (OHpak SB-806 M HQ; Shodex, Tokyo, Japan) and reflective index detector (RID-10A; Shimadzu), as described previously [36].

### Photosynthesis analysis

Oxygen exchange was monitored with an oxygen electrode (Hansatech, King’s Lynn, UK). For the measurement, a reaction mixture (2 mL) containing 50 mM HEPES-KOH (pH 7.6), 10 mM NaHCO_3_ and an arbitrary amount of cells (10 μg chlorophyll mL^-1^) was first incubated in the dark for 10 min under air-equilibrated conditions and was then illuminated with actinic light (red light of wavelength >640 nm) at the indicated light intensity at 25°C. During the measurements, the reaction mixture was mixed with a magnetically controlled micro-stirrer.

## Additional file

Additional file 1:
**Concentration of cellular biomass cultivated under 120 μmol photons m** ^**-2**^ **s** ^**-1**^ **light intensity and 1% CO**_**2**_ **conditions.** Difference in biomass concentration was statistically analyzed by one-way ANOVA. A *post hoc* Tukey’s Honestly Significant Difference test was carried out on the grouped means. Values are the averages from measurements of ten different cell cultures, ±SD. Values followed by the same letter are not significantly different (*P* > 0.01).

## Abbreviations

AcCoA: acetyl-CoA
ADP-Glc: ADP-glucose
AEF: alternative electron flow
CE: capillary electrophoresis
DCW: dry cell weight
Flv: flavodiiron
F6P: fructose-6-phosphate
G1P: glucose-1-phosphate
G6P: glucose-6-phosphate
GT: glucose-tolerant
LEF: linear electron flow
MS: mass spectrometry
PEP: phospho*enol*pyruvate
2PGA: 2-phosphoglycerate
3PGA: 3-phosphoglycerate
PTFE: polytetrafluoroethylene
RuBP: ribulose-1,5-bisphosphate
S7P: sedoheptulose-7-phosphate
VC: vector control
WWC: water-water cycle
