# Supplementary material for: Overexpression of flv3 improves photosynthesis in the cyanobacterium Synechocystis sp. PCC6803 by enhancement of alternative electron flow
Source: Biotechnol Biofuels. 2014 Dec 31;7:493. doi: 10.1186/s13068-014-0183-x (PMC4300077; doi:10.1186/s13068-014-0183-x)
Supplement: Additional file 1: — Concentration of cellular biomass cultivated under 120 μmol photons m -2 s -1 light intensity and 1% CO 2 conditions. Difference in biomass concentration was statistically analyzed by one-way ANOVA. A post hoc Tukey’s Honestly Significant Difference test was carried out on the grouped means. Values are the averages from measurements of ten different cell cultures, ±SD. Values followed by the same letter are not significantly different (P > 0.01). [file 13068_2014_183_MOESM1_ESM.pdf]

Additional file 1 - Concentration of cellular biomass cultivated under 120 μmol photons m^{-2} s^{-1} light intensity and 1% CO_2 conditions.

Time (day)	Biomass (g-DCW L^{-1})		
	GT	Flv3ox	VC
3	0.95 ± 0.07^{ab}	1.07 ± 0.14^{a}	0.82 ± 0.032^{b}
4	1.59 ± 0.21^{a}	2.01 ± 0.22^{b}	1.42 ± 0.06^{a}
5	2.09 ± 0.12^{a}	2.46 ± 0.07^{b}	2.17 ± 0.07^{a}
6	2.30 ± 0.09^{a}	3.11 ± 0.26^{b}	2.50 ± 0.05^{a}
7	2.48 ± 0.20^{a}	3.20 ± 0.18^{b}	2.74 ± 0.20^{a}

Difference in biomass concentration was statistically analysed by one-way ANOVA. A *post hoc* Tukey's Honestly Significant Difference test was carried out on the grouped means. Values are the averages from measurements of ten different cell cultures, ±SD. Values followed by the same letter are not significantly different ($P > 0.01$).
